# Adjustment of spurious correlations in co-expression measurements from RNA-Sequencing data

**DOI:** 10.1093/bioinformatics/btad610

**Published:** 2023-10-06

**Authors:** Ping-Han Hsieh, Camila Miranda Lopes-Ramos, Manuela Zucknick, Geir Kjetil Sandve, Kimberly Glass, Marieke Lydia Kuijjer

**Affiliations:** Centre for Molecular Medicine Norway (NCMM), Nordic EMBL Partnership, University of Oslo, Oslo 0318, Norway; Department of Informatics, University of Oslo, Oslo 0316, Norway; Department of Biostatistics, Harvard T.H. Chan School of Public Health, Boston, MA 02115, United States; Department of Medicine, Harvard Medical School, Boston, MA 02115, USA; Channing Division of Network Medicine, Brigham and Women's Hospital, Boston, MA 02115, United States; Oslo Centre for Biostatistics and Epidemiology, Institute of Basic Medical Sciences, University of Oslo, Oslo 0317, Norway; Department of Informatics, University of Oslo, Oslo 0316, Norway; Department of Biostatistics, Harvard T.H. Chan School of Public Health, Boston, MA 02115, United States; Channing Division of Network Medicine, Brigham and Women's Hospital, Boston, MA 02115, United States; Centre for Molecular Medicine Norway (NCMM), Nordic EMBL Partnership, University of Oslo, Oslo 0318, Norway; Department of Pathology, Leiden University Medical Center, Leiden 2300RC, The Netherlands; Leiden Center of Computational Oncology, Leiden University Medical Center,Leiden 2300RC, The Netherlands

## Abstract

**Motivation:**

Gene co-expression measurements are widely used in computational biology to identify coordinated expression patterns across a group of samples. Coordinated expression of genes may indicate that they are controlled by the same transcriptional regulatory program, or involved in common biological processes. Gene co-expression is generally estimated from RNA-Sequencing data, which are commonly normalized to remove technical variability. Here, we demonstrate that certain normalization methods, in particular quantile-based methods, can introduce false-positive associations between genes. These false-positive associations can consequently hamper downstream co-expression network analysis. Quantile-based normalization can, however, be extremely powerful. In particular, when preprocessing large-scale heterogeneous data, quantile-based normalization methods such as smooth quantile normalization can be applied to remove technical variability while maintaining global differences in expression for samples with different biological attributes.

**Results:**

We developed SNAIL (Smooth-quantile Normalization Adaptation for the Inference of co-expression Links), a normalization method based on smooth quantile normalization specifically designed for modeling of co-expression measurements. We show that SNAIL avoids formation of false-positive associations in co-expression as well as in downstream network analyses. Using SNAIL, one can avoid arbitrary gene filtering and retain associations to genes that only express in small subgroups of samples. This highlights the method’s potential future impact on network modeling and other association-based approaches in large-scale heterogeneous data.

**Availability and implementation:**

The implementation of the SNAIL algorithm and code to reproduce the analyses described in this work can be found in the GitHub repository https://github.com/kuijjerlab/PySNAIL.

## 1 Introduction

Understanding the cell’s regulatory machinery can provide relevant insights into healthy tissues as well as human diseases (Boyle *et al.* 2017, Sonawane *et al.* 2017). While certain experimental techniques, including chromatin immunoprecipitation sequencing (ChIP-Seq), can map interactions made by regulatory elements, it is challenging to directly observe the combined effect of multiple regulators in a systematic way. Previous studies have shown that genes undergoing similar regulatory processes tend to have coordinated expression, also called “co-expression,” across samples (Marco *et al.* 2009, Gu *et al.* 2011, Guo *et al.* 2016). Therefore, estimates of gene co-expression are commonly used to infer associations between genes. Gene co-expression can also be used in combination with other molecular data to improve the detection of regulatory interactions (Glass *et al.* 2013, Nicolle *et al.* 2015, Petralia *et al.* 2015, Reiss *et al.* 2015, Kuijjer *et al.* 2020).

Most commonly, co-expressed genes are identified using Pearson correlation, Spearman correlation (Langfelder and Horvath 2008), or mutual information (Meyer *et al.* 2007, Lachmann *et al.* 2016). Another popular approach is to construct regression models that predict the expression of one gene based on the expression of all other genes or potential regulators, and then apply variable selection to identify dependencies between genes (Irrthum *et al.* 2010, Haury *et al.* 2012). Both types of approaches aim to identify associations between genes based on their coordinated expression levels across all samples in a dataset. Therefore, as with standard gene expression analysis, it is essential to preprocess the expression data that is used as input for co-expression analysis (Silverman *et al.* 2020).

To correct for technical variability across samples, various RNA-Sequencing (RNA-Seq) normalization methods have been developed (Anders and Huber 2010, Robinson and Oshlack 2010). Since biological and technical variability cannot be distinguished in RNA-Seq data, algorithmic modeling is required to infer technical variability and correct the read counts for the latter. Most normalization methods correct for technical variability using global properties (statistics that consider every sample). For instance, relative log expression (RLE) normalization, as used in DESeq, computes the median ratio of gene counts relative to the geometric mean across all samples (Anders and Huber 2010). Without providing information on what specific biological group a sample belongs to, global shifts in gene expression caused by biological differences may be removed during the normalization process (Evans *et al.* 2018). To address this issue, a quantile normalization-based method was recently developed that utilizes the information of the experimental design provided by the user to categorize samples into one or more biologically meaningful groups. Both group-specific and global properties of the expression distribution are then used to correct for technical variability. This method, called smooth quantile normalization, or *qsmooth*, yields better preservation of global shifts in expression as well as adequate control over the variability between distributions within groups (Hicks *et al.* 2018). While *qsmooth* was only recently developed, it has already been used in several analyses with large heterogeneous RNA-Seq datasets (Sonawane *et al.* 2017, Tosti *et al.* 2018, Anderson *et al.* 2019, Zhao *et al.* 2021).

**Figure 1.**
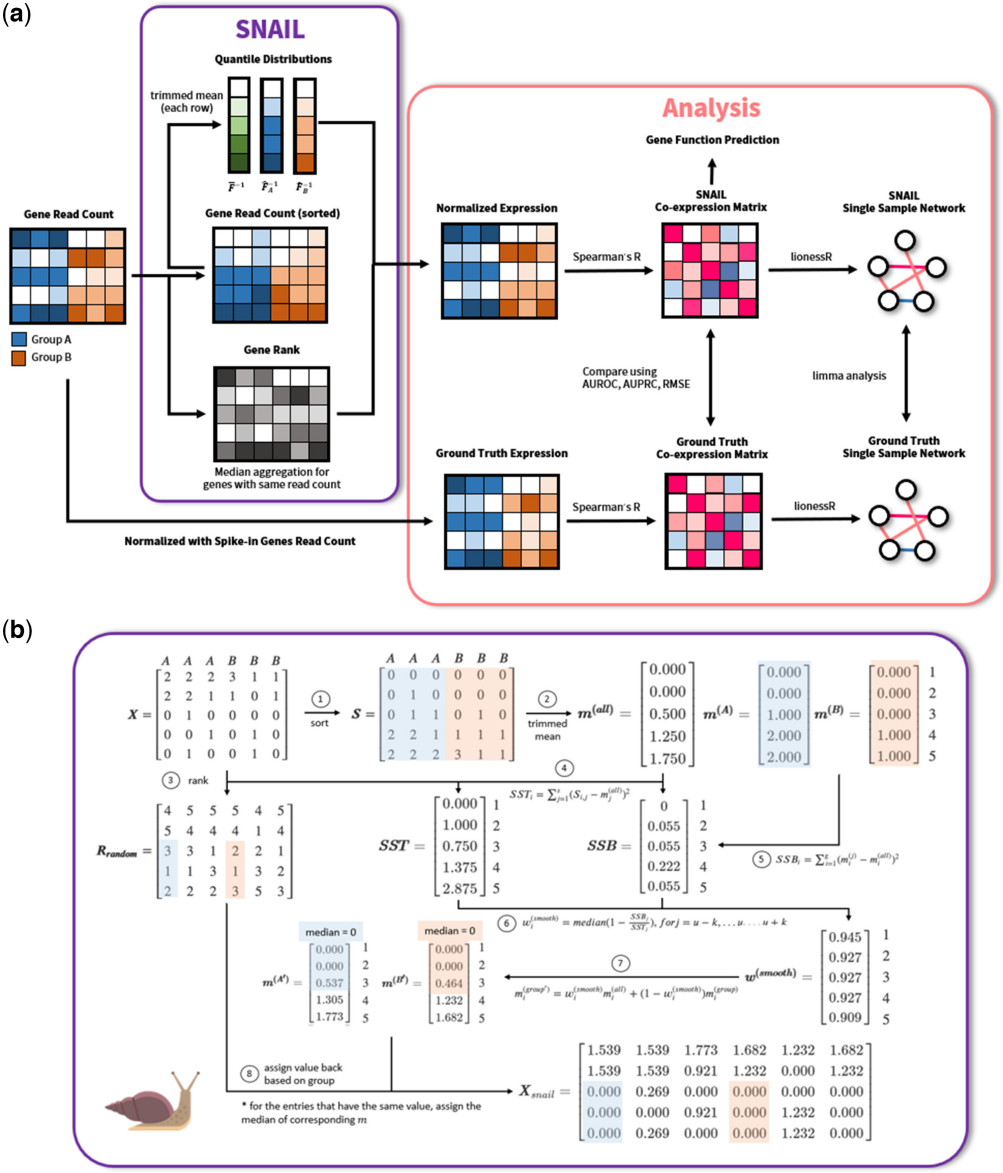
Schematic overview of (a) SNAIL and the analyses performed in this work. (b) SNAIL algorithm. SNAIL is based on smooth quantile normalization but uses the trimmed mean to derive the quantile distribution for all samples as well as for every biological group of samples. In addition, SNAIL uses the median of the quantiles to normalize the expression for genes with the same read count in one sample.

Here, we show that quantile-based normalization methods, and in particular smooth quantile normalization, can introduce false-positive associations between genes. We found that this can particularly occur in datasets that have large differences in the library size across samples. To correct for these false-positives, we developed SNAIL, or Smooth-quantile Normalization Adaptation for the Inference of co-expression Links. SNAIL is a modified implementation of smooth quantile normalization which uses a trimmed mean to determine the quantile distribution and applies median aggregation for genes with shared read counts (Fig. 1). We analyzed RNA-Seq data from the Genotype-Tissue Expression (GTEx) Consortium (Ardlie *et al.* 2015) to showcase the problem, and data from the Mouse Encyclopedia of DNA Elements (ENCODE) (Stamatoyannopoulos *et al.* 2012) to validate the method. We found that SNAIL effectively removes false-positive associations between genes, without the need to select an arbitrary threshold or to exclude genes from the analysis. We anticipate that our method will benefit future co-expression and regulatory network analyses, in particular those that involve the analyses of large-scale heterogeneous RNA-Seq datasets.

## 2 Materials and methods

### 2.1 Preparation of GTEx data

We downloaded RNA-Seq count data from the GTEx Consortium V8 release (Ardlie *et al.* 2015). We followed the procedure conducted by Paulson *et al.* (2017) to merge tissues with similar expression profiles. We selected those tissues previously reported to have the largest number of genes that deviate the most when comparing the expression in the tissue-of-interest with the median expression across all tissues—*testis*, *kidney—cortex*, *brain—other*, *breast—mammary tissue*, and *whole blood* (Sonawane *et al.* 2017). Note that the *brain—other tissue* consists of several merged brain regions, as described in Paulson *et al.* (2017). The resulting datasets consisted of 1575 samples and 55 878 genes. Combined with the tissue information (*biosample_name*) in the meta data, we then used the Bioconductor package *qsmooth* (version 1.4.0) (Hicks *et al.* 2018) to perform smooth quantile normalization, using tissue as the user-defined “sample group” for calculating the *group reference distributions* (see Section 3.1 for a more detailed explanation of smooth quantile normalization). Finally, we applied our SNAIL method to the same dataset, again using tissue as the user-defined “sample group.”

### 2.2 Preparation of ENCODE data

For the validation datasets, we downloaded bulk polyA plus RNA-seq count data consisting of twelve tissues (*embryonic facial prominence*, *forebrain*, *heart*, *hindbrain*, *intestine*, *kidney*, *limb*, *liver*, *lung*, *midbrain*, *neural tube*, *stomach*) from the Mouse ENCODE database using the Bioconductor package *ENCODExplorer* (version 2.14.0, download date September 11, 2020) (Beauparlant *et al.* 2015). Among all the available experiments, we extracted those for which External RNA Controls Consortium (ERCC)-only spike-ins (accession: ENCSR884LPM) information was available. The resulting datasets consisted of 126 samples and 43 346 genes.

To establish the validation dataset, we normalized the read counts by the expression of 96 spike-in genes (Supplementary Section S4). Similar to the preparation of GTEx dataset (Section 2.1), we used both *qsmooth* and SNAIL to perform normalization on the original count data. The corresponding tissue information of each sample (*SMTSD*) was then used as the “sample group” for tissue-aware normalization.

### 2.3 Definition of tissue exclusive genes

For both the GTEx and ENCODE datasets, we extracted the genes that were exclusively expressed in one tissue, denoted as *tissue-exclusive genes*. We define tissue-exclusivity using the following two criteria: (i) the median ground truth expression of the gene is higher than or equal to 10 across all samples from the tissue of interest, and (ii) the median ground truth expression is lower than or equal to 1 across samples from all other tissues. Note that we used these criteria to facilitate the visualization of the problem. We did not look into the biological role of these genes in this study.

To showcase the false-positive associations introduced by smooth quantile normalization, we compared the Spearman’s rank correlation coefficients for these tissue-exclusive genes based on *qsmooth*-normalized and SNAIL-normalized expression levels. Since the number of tissue-exclusive genes varies drastically across different tissues, when visualizing the issue we only retained tissue-exclusive genes of tissues with 5–1000 tissue-exclusive genes; genes exclusively expressed in testis for the GTEx dataset, and embryonic facial prominence, limb, neural tube, and forebrain for the ENCODE dataset were thus excluded from the visualization. Note that the exclusion of these genes is not required when applying SNAIL. The numbers of tissue-exclusive genes for the two datasets are shown in Supplementary Section S5.

### 2.4 Evaluation of the SNAIL method

To evaluate the performance of SNAIL, we applied two different strategies to the data obtained from GTEx and from ENCODE. For the GTEx dataset, we defined two genes to be associated if (i) the two genes were both expressed exclusively in the same tissue or if (ii) the two genes shared the same functional annotation. To extract the functional annotation of each gene, we used the get_functional_annotation function provided in the stringdb package (Szklarczyk *et al.* 2019). Including this information allowed us to identify false-positive associations between genes when no validation data is present.

For the ENCODE dataset, we defined two genes to be associated when the absolute value of their Spearman’s rank correlation coefficient, based on the ground truth expression, was higher than or equal to a specific value, ranging from 0.2 to 0.8. This allowed us to evaluate the performance of SNAIL under different strengths of ground truth associations between genes.

### 2.5 Downstream network analyses

We performed three downstream co-expression network analyses to evaluate whether the false-positive associations can propagate through downstream network analysis.

First, we performed network comparisons on sample-specific networks. We constructed sample-specific networks using Bioconductor package *lionessR* (version 1.2.0-0) (Kuijjer *et al.* 2019a) with Spearman’s rank correlation coefficients as the network reconstruction function. *lionessR* is based on the LIONESS algorithm (Kuijjer *et al.* 2019b), which assumes that edges estimated in an “aggregate” network model are a linear combination of edges specific to each of the input samples. This allows for the estimation of individual sample edge weights using a linear equation. These edge weights can then be used for sample-specific network analysis, as done previously (Lopes-Ramos *et al.* 2018, 2020, 2021). We modeled these networks with the *qsmooth*-normalized expression data as input, as well as based on the SNAIL-normalized expression data, so that we had two collections of networks that we could compare. We then used the Bioconductor package *limma* (version 3.44.1) (Ritchie *et al.* 2015) to identify significant differences in the distributions of edge weights across the constructed sample-specific networks versus the ground truth co-expression networks. Note that, although the standard application of *limma* is to test for differential expression, the authors of the method suggest that *limma’*s linear modeling strategy can be used for other applications beyond gene expression. Here, we performed differential edge analysis and posterior variance estimation based on the sample groups using the function *lmfit* and *eBayes* from the *limma* package.

Next, we performed hub gene identification on networks representing each tissue. To explore this, we followed the procedure described in Lopes-Ramos *et al.* (2021) to aggregate sample-specific networks inferred with the LIONESS algorithm into tissue-specific networks. The hub score of each gene in these networks was then computed using the HITS algorithm (Kleinberg 1999) using the *hits* function provided in *networkx* package (Hagberg *et al.* 2008).

Lastly, we performed gene function prediction based on the co-expression values, following the procedure presented in Hew *et al.* (2020). To evaluate whether multiple functions of genes can be correctly predicted, we made an adaptation that uses the Jaccard Index instead of the F1 score. We predicted two genes to be associated if the Spearman’s rank correlation coefficient between them was higher than or equal to a specific value, ranging from 0.1 to 0.8. Thereafter, we predicted the function of tissue-exclusive genes if more than a specific proportion (ranging from 0.01 to 0.4) of the co-expressed genes shared the same functional annotation obtained from KEGG pathways (Kanehisa *et al.* 2016).

Note that for the above-mentioned analyses, we excluded 1848 tissue-exclusive genes for GTEx and one tissue-exclusive gene for ENCODE that had either zero or multiple gene symbol annotations, based on the annotation obtained with the *Biomart.query* function provided by GSEApy package (version 1.0.4) (Durinck *et al.* 2009).

### 2.6 Code availability

The implementation of the SNAIL algorithm and all of the analyses conducted in this study can be reproduced using the Snakemake workflow management system (Mölder *et al.* 2021) from the GitHub repository https://github.com/kuijjerlab/PySNAIL.

## 3 Results

### 3.1 Quantile-based normalization methods can introduce false-positive associations in large-scale heterogeneous datasets

In this section, we demonstrate how quantile-based normalization—and in particular smooth quantile normalization can introduce false-positive associations between genes. To do so, we will present a case study on co-expression analysis for genes that are exclusively expressed in a specific tissue. We used RNA-Seq data from the Genotype Tissue Expression (GTEx) project (Ardlie *et al.* 2015) and selected the tissues with high levels of tissue-specific gene expression (see Section 2.3). We performed smooth quantile normalization to remove the technical variability presented in the dataset, while preserving the global expression differences between the different tissues. Next, we extracted the tissue-exclusive genes for each tissue (see Section 2.3) and performed co-expression analysis using Spearman’s rank correlation coefficient (ρ).

**Figure 2.**
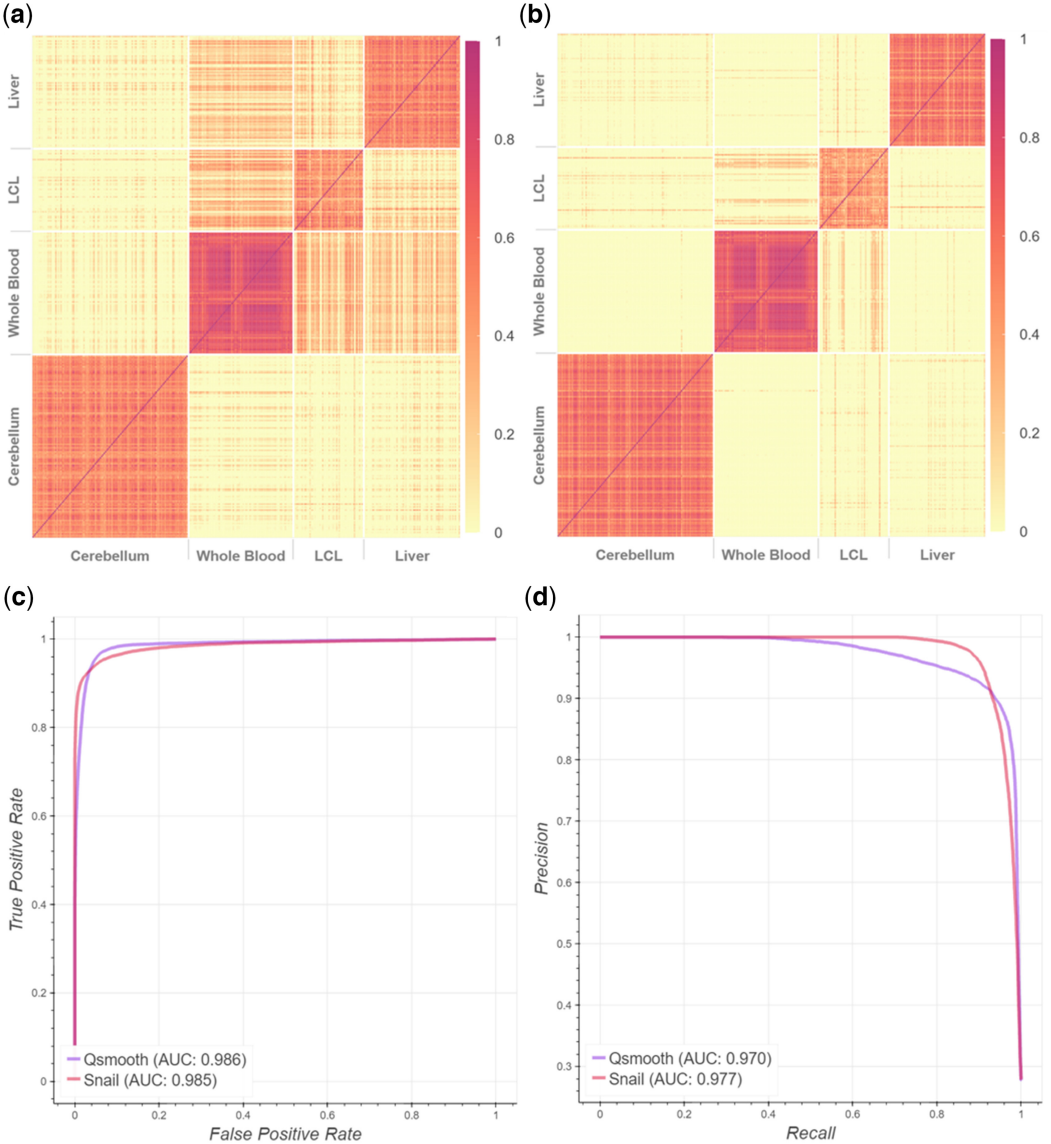
Spearman’s rank correlation coefficients between tissue-exclusive gene pairs, based on smooth quantile normalized data from GTEx. (a) False-positive associations are detected in qsmooth-normalized data between tissue-exclusive genes from different tissues, as can be seen in off-diagonal blocks of expression. (b) SNAIL removes most of these false-positive associations. (c) The receiver operator curve and (d) precision–recall curve, where the ground truth association between genes is defined by whether the genes share the same functional annotation in the STRING database.

We expect to observe co-expression between pairs of genes that are both expressed in the same tissue, but not between pairs of genes, each of which is exclusively expressed in a different tissue. However, while we do observe high co-expression levels between tissue-exclusive genes in the same tissue, we also observe relatively high levels of co-expression between pairs of genes that are exclusively expressed in different tissues. In particular, we observe such associations between *whole blood*, *lymphoblastoid cell lines (LCL)* and *liver* (Fig. 2a).

To understand how these false-positive associations arise, we dissected both quantile normalization (Supplementary Sections S1 and S2) and the smooth quantile normalization algorithm as it is implemented in the Bioconductor package *qsmooth* (Hicks *et al.* 2018). We found that this problem is more prone to arise with smooth quantile normalization. Therefore, we focus on this methodology in the remainder of this manuscript.

*Qsmooth* computes the average expression level in each quantile, considering only the samples within a given user-defined group—the *group reference distribution*—as well as the average expression level in each quantile, considering all samples—the *background reference distribution*. The method then estimates the empirical reference distribution to be the weighted average of the background reference distribution and the group reference distribution, where the weight coefficient is computed based on the proportion of explained variability in the group quantile distribution. Since *qsmooth* uses the average to derive the quantile distributions, the values corresponding to small quantiles can be nonzero, despite the fact that most of the values that those quantiles are based on are zeros (see also Supplementary Sections S1 and S2).

Another important detail of *qsmooth* is the ranking method used to process genes that have the same read count (tied counts) in each sample. The quantiles corresponding to these genes are dependent on the number of genes that have the same read count in that specific sample (Supplementary Sections S1 and S2). Therefore, even if a gene would have the exact same read count in two different samples, the corresponding quantiles can be drastically different. Especially for zero-inflated RNA-Seq data in heterogeneous datasets that have large differences between the smallest and the largest number of nonexpressed genes across samples, lowly expressed genes could share the same quantile with nonexpressed genes in different samples. As the normalized values are dependent on the quantiles of the expression distribution in each sample, this can introduce small technical variability across samples, which consequently can lead to false-positive correlation coefficients. This issue is more prevalent between, for example, genes that are only expressed in a subset of samples (Supplementary Sections S1 and S2).

### 3.2 Smooth quantile Normalization Adaptation for the Inference of co-expression Links

It is important to be able to take advantage of smooth quantile normalization, so that one can explicitly model the biological variability and retain global expression differences in heterogeneous data. However, we also need to ensure the identified co-expression signals are reliable. This motivated us to develop SNAIL (Fig. 1), an adaptation of smooth quantile normalization. Instead of using the average of the observed quantile distributions, SNAIL uses the trimmed mean (customizable; by default SNAIL trims the 15% largest and smallest values) to infer the heuristic reference quantile distribution. In addition, when normalizing genes with the same read count, SNAIL uses median aggregation of the corresponding quantiles to substitute the original data with the normalized values (Supplementary Section S3). As we show below, these adaptations drastically reduce the formation of false-positive associations.

Aside from the above-mentioned adaptation, we implemented a diagnostic function that computes the proportion of affected genes for each sample. This utility can help detect whether regular smooth quantile normalization would introduce false-positive associations between genes in a specific dataset (Supplementary Section S1).

### 3.3 SNAIL reduces false-positive associations

We applied SNAIL to normalize gene expression levels in the GTEx data and repeated the co-expression analysis described above using the same set of genes and tissues (see also Section 2.4). Comparing the Spearman’s rank correlation coefficients obtained in the *qsmooth*- and SNAIL-normalized data, we found that SNAIL is capable of removing false-positive associations, while modeling tissue-exclusive biological variability similarly to smooth quantile normalization (Fig. 2a and b). With the above-mentioned threshold of ρ= 0.3 to define co-expression, SNAIL reduces the number of such false-positive associations from 3442 (8.6%) to 231 (0.58%). Note that the threshold to define co-expression based on the Bonferroni-adjusted *P*-value is 0.117 in this case (the adjusted *P*-value for each gene pair is shown in Supplementary Section S6).

After normalizing the data with SNAIL, we found that some associations remained between genes that were exclusively expressed in different tissues, including genes exclusively expressed in LCL and whole blood, LCL and liver, and LCL and cerebellum. We cautiously conclude that these associations are not introduced by smooth quantile-based normalization, but are present because of our definition of tissue exclusivity, as we observed similar results for the nonnormalized count data. Next, we defined ground truth association between genes if two genes shared the same functional annotation in the STRING database. This allowed us to identify false-positive associations without the spike-in validation dataset. This experiment shows that SNAIL normalization yields higher precision compared to normalization with *qsmooth* (Fig. 2c and d).

To better quantify the capability of SNAIL to reduce these false-positive associations under different strengths of ground truth associations, we applied the normalization method to RNA-Seq data from the Mouse ENCODE database, which includes spike-ins (Section 2.2). We used the expression of spike-in genes to normalize the read count (Supplementary Section S4), creating the ground truth expression dataset. Comparing Spearman’s rank correlation coefficients obtained with *qsmooth* and SNAIL with those derived from the ground truth expression dataset for each gene pair, we observed that the root mean square error (RMSE) between the correlation coefficients decreases from 0.03856 to 0.01516 after applying SNAIL.

**Figure 3.**
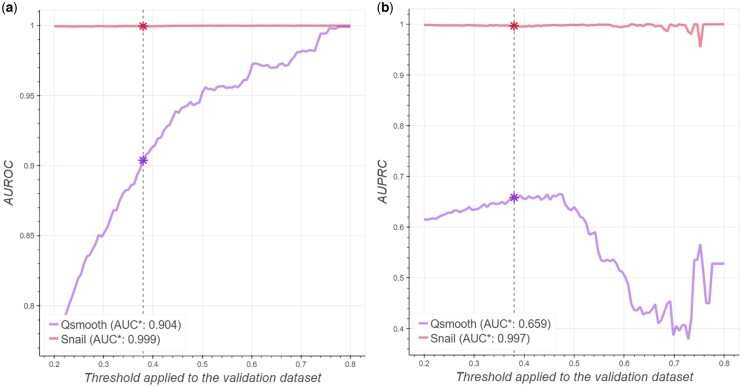
SNAIL can effectively reduce the number of false-positive associations in co-expression analysis. The *x*-axis denotes the threshold of absolute Spearman’s rank correlation coefficient based on ground truth expression that defines true associations between genes, while the *y*-axis corresponds to the area under the receiver operator curve (AUROC, panel a) and precision–recall curve (AUPRC, panel b). The vertical dashed line indicates the threshold of a significant association based on the Bonferroni adjusted *P*-value (0.379). AUC* specified in the legend indicates the area under the curve using that threshold of significant association.

We next conducted receiver–operator curve and precision–recall curve analyses and reported the area under the two curves. We found that SNAIL can reduce the false-discovery rate in co-expression analysis, regardless of the strength of the correlation signal (Fig. 3). Note that when the true association is more strictly defined (correlation coefficient above 0.7), the small number of positive associations (<30 positive associations) causes a fluctuation in the AUPRC. In addition, we evaluated different cutoffs for the trimmed mean used in SNAIL, and found that its performance is consistent across different cutoff values (Supplementary Section S7).

In addition to these analyses, we compared SNAIL’s performance to that of other commonly used normalization methods, such as RLE and transcripts per million (TMM) (Supplementary Section S8). Compared to *qsmooth*, SNAIL effectively removes false-positive associations while reaching a similar performance in detecting correct associations between genes as RLE and TMM (Supplementary Fig. S8). Note however that the performance of these methods cannot be directly compared since SNAIL and smooth quantile normalization explicitly model the global differences across different biological groups and show better control for the variability between distributions within groups (Supplementary Fig. S9). The comparisons we made here aim to showcase the limitation of the original implementation of smooth quantile normalization when normalizing data to be used in correlation analyses.

### 3.4 SNAIL improves downstream network analyses

**Figure 4.**
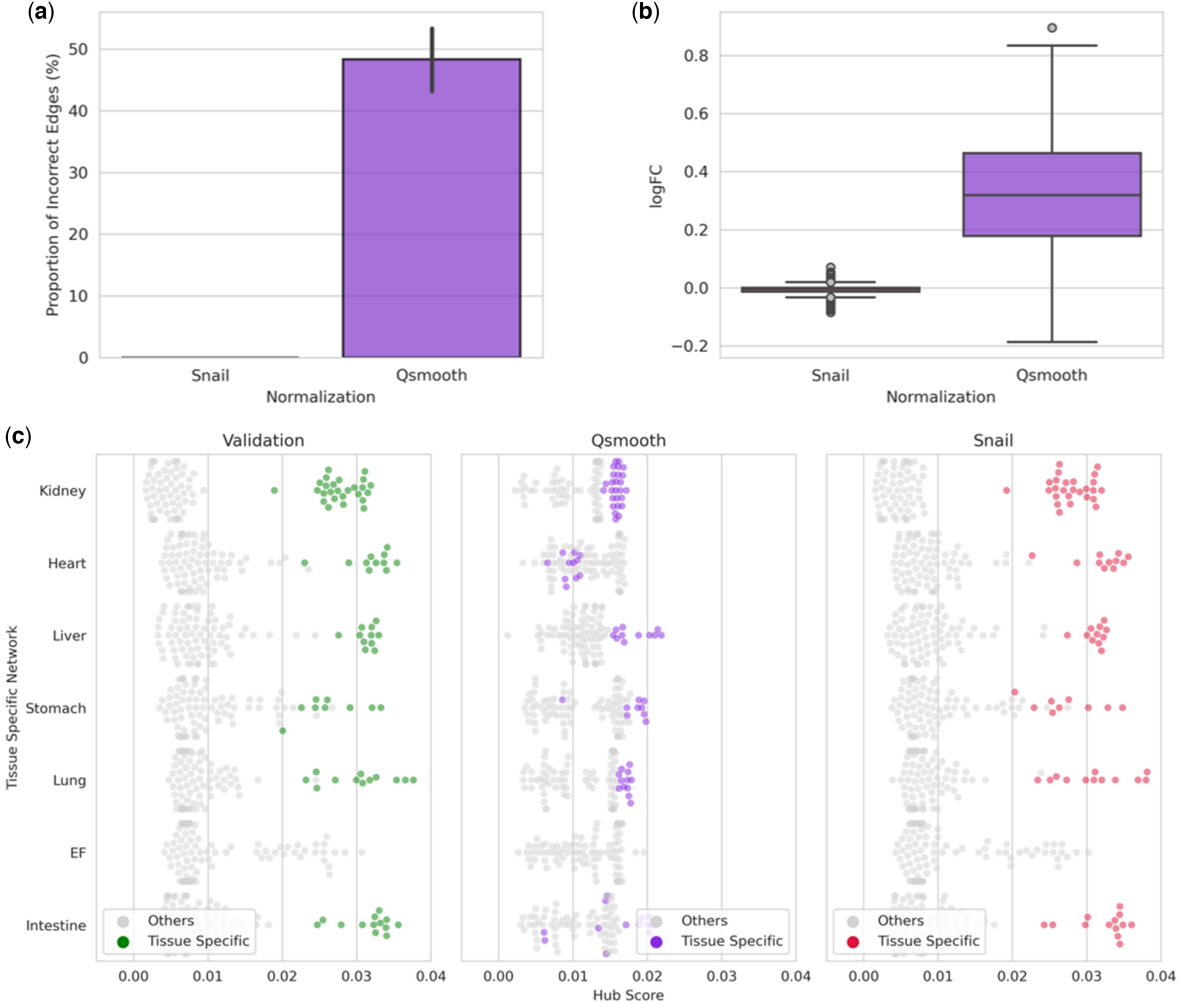
Evaluation of downstream network analyses of ENCODE dataset. (a) The proportion of incorrect edges of each gene in the sample-specific networks. (b) Log-transformed fold changes on the mean value (computed across sample-specific networks) of the edge weights comparing the network constructed on ground truth expression with the networks. (c) Hub scores of tissue-exclusive genes in the tissue-specific networks, where each point represents a gene, and the genes exclusively expressed in the corresponding tissue are colored.

We next wanted to evaluate whether the false-positive associations introduced by quantile-normalized methods also affect downstream network analysis. We first built sample-specific networks using the LIONESS algorithm (Section 2.5). Then, we compared the distribution of edge weights across all sample-specific networks constructed on ground truth co-expression to the distribution of edge weights from the co-expression networks constructed on (i) *qsmooth*- and on (ii) SNAIL-normalized expression, using a *t*-test for each gene pair independently. Figure 4 shows that the false-positive associations propagate through downstream network analysis, creating 1871 false-positive edges from a total of 3828 potential edges between genes exclusively expressed in different tissues. In SNAIL-normalized data, no edge weight significantly differs from the network built on the ground truth expression (FDRadjusted *P*-value ≤ 0.05, Fig. 4a and 4b).

Since the LIONESS algorithm applied to Spearman’s rank correlation coefficient computes the contribution of the association of each gene pair to the background network model, we expect the tissue-exclusive genes would be the main contributing factor to the network modeled for a specific tissue. This can be confirmed by the validation dataset from ENCODE (Fig. 4c). The majority of hub scores for tissue-exclusive genes from the networks constructed on SNAIL-normalized data are higher than the ones constructed on *qsmooth*-normalized data (Fig. 4c, Supplementary Section S9). The scores obtained with SNAIL are close to those obtained from the networks constructed on the validation dataset (RMSE: 0.018). These results show that SNAIL-normalized gene expression can preserve biological signals in downstream network analysis.

**Figure 5.**
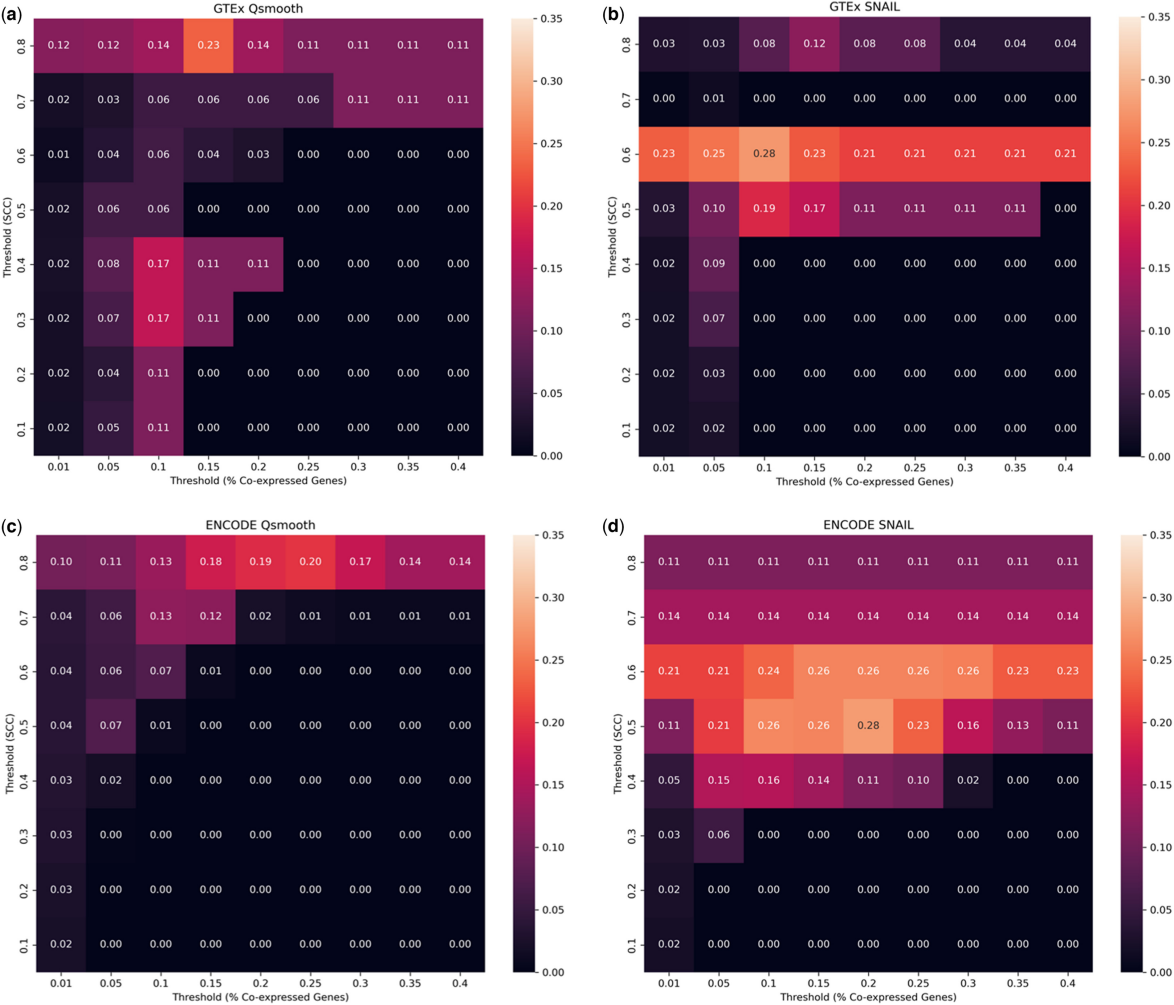
Evaluation of gene prediction using co-expression on (a) GTEx Qsmooth, (b) GTEx SNAIL, (c) ENCODE Qsmooth, and (d) ENCODE SNAIL normalized expression. The heatmaps indicate the Jaccard index of the predicted gene function based on different Spearman’s correlation thresholds and proportions of co-expressed genes that shared the same functional annotation.

Lastly, we performed gene function prediction based on gene co-expression, following a previously published procedure [Hew *et al.* (2020), see Section 2.5]. We found that SNAIL outperforms *qsmooth*, resulting in a higher average Jaccard index between the ground truth and the predicted gene function (Fig. 5). Note that function prediction of tissue-exclusive genes based on *qsmooth* improves when a stricter threshold is applied to define the association between genes (Spearman’s correlation coefficients ≥ 0.8). This indicates that the false-positive associations introduced by *qsmooth* are detrimental to gene function prediction. However, some associations between genes that share similar functions are preserved if there is a strong association.

## 4 Discussion

Here, we showed that the application of quantile-based normalization approaches, and specifically, smooth quantile normalization, to RNA-Seq data can introduce false-positive associations between genes, and that this can propagate to and affect downstream network analyses. We found that false-positive associations particularly arise when there is a large difference between the smallest and the largest number of nonexpressed genes across the samples in the dataset. This can, for example, occur when dealing with RNA-Seq datasets collected from large-scale projects that include heterogeneous data. For instance, data from The Cancer Genome Atlas (Weinstein *et al.* 2013), ENCODE, and GTEx, which have previously been used by various groups to conduct co-expression, or co-expression-based, network analysis (Ardlie *et al.* 2015, Pierson *et al.* 2015, Saha *et al.* 2017, Sonawane *et al.* 2017, Lopes-Ramos *et al.* 2020).

A frequently applied strategy that attempts to remove potential false-positive associations is filtering out genes with low read counts across a certain number of samples. However, thresholds used for filtering are often chosen arbitrarily, and can remove genes that are specifically expressed in a subset of samples, such as the tissue-exclusive genes that we described in our example network analysis in GTEx data. Therefore, arbitrary filtering is not ideal if one aims to compare gene associations or networks derived from different subgroups of samples. Moreover, it would be ideal to include all genes in large-scale network analysis, as certain network reconstruction algorithms make use of the entire distribution of gene expression and thus filtering out genes may remove some signal from the input dataset (Glass *et al.* 2013).

SNAIL retains global expression differences in heterogeneous datasets through the use of both group-specific and global properties. This allows direct comparison of networks modeled on heterogeneous datasets. Note that SNAIL requires information on the biological group samples belong to, and therefore is not applicable to samples without annotation. In addition, as SNAIL uses the trimmed mean to infer the quantile distribution, the latter may not be inferred correctly if the number of samples in biological groups is limited. We therefore recommend applying SNAIL to large heterogeneous datasets with a sufficient amount of samples to estimate the reference quantile distributions for each group based on the trimmed mean.

We show that SNAIL avoids the formation of false-positive associations introduced by smooth quantile normalization. By using the trimmed mean to infer the reference quantile distribution as well as median aggregation for genes with the same read count, SNAIL avoids the formation of false-positive associations. Importantly, as the method does not require gene filtering, it allows for direct comparison of networks modeled on heterogeneous datasets.

While we specifically focused our examples on modeling co-expression across different tissues, false-positive associations can also arise when comparing other biological conditions that show large differences in expression profiles under certain experimental settings, such as when comparing networks for males and females Lopes-Ramos *et al.* (2020). We also envision that other methods that are based on correlations, such as eQTL studies, could potentially include quantile-based normalization-introduced false-positives, and could benefit from normalization with SNAIL. In general, we would like to raise awareness of implementing tools designed for gene expression data in existing correlation-based approaches or pipelines. Most of the published evaluations of normalization methods are based on comparing differences between ground truth and normalized expression levels. However, the impact of normalization on correlation-based measures is often neglected.

Heterogeneous datasets with increasing numbers of samples and conditions will likely be published in the near future, and new methods for combining data from different studies (Collado-Torres *et al.* 2017) will result in the emergence of even larger and more heterogeneous datasets. As these datasets will become available for analysis, we expect SNAIL to become an important tool that will allow for more precise analyses of large-scale data with network-based approaches.

## Supplementary Material

btad610_Supplementary_Data

## References

[btad610-B1] Anders S , Huber W. Differential expression analysis for sequence count data. Nat Prec 2010;11:R106. 10.1186/gb-2010-11-10-r106 PMC3218662 20979621

[btad610-B2] Anderson D , Baynam G, Blackwell JM et al Personalised analytics for rare disease diagnostics. Nat Commun 2019;10:5274–8. 31754101 10.1038/s41467-019-13345-5 PMC6872807

[btad610-B3] Ardlie KG , Deluca DS, Segrè AV et al; The GTEx Consortium. The genotype-tissue expression (GTEx) pilot analysis: multitissue gene regulation in humans. Science 2015;348:648–60. 25954001 10.1126/science.1262110 PMC4547484

[btad610-B4] Beauparlant C , Lemacon A, Droit A. Encodexplorer: a compilation of encode metadata. R Package Version 2015;1.

[btad610-B5] Boyle EA , Li YI, Pritchard JK. An expanded view of complex traits: from polygenic to omnigenic. Cell 2017;169:1177–86. 28622505 10.1016/j.cell.2017.05.038 PMC5536862

[btad610-B6] Collado-Torres L , Nellore A, Kammers K et al Reproducible RNA-seq analysis using recount2. Nat Biotechnol 2017;35:319–21. 28398307 10.1038/nbt.3838 PMC6742427

[btad610-B7] Durinck S , Spellman PT, Birney E et al Mapping identifiers for the integration of genomic datasets with the R/bioconductor package biomart. Nat Protoc 2009;4:1184–91. 19617889 10.1038/nprot.2009.97 PMC3159387

[btad610-B8] Evans C , Hardin J, Stoebel DM. Selecting between-sample RNA-seq normalization methods from the perspective of their assumptions. Brief Bioinform 2018;19:776–92. 28334202 10.1093/bib/bbx008 PMC6171491

[btad610-B9] Glass K , Huttenhower C, Quackenbush J et al Passing messages between biological networks to refine predicted interactions. PLoS One 2013;8:e64832. 23741402 10.1371/journal.pone.0064832 PMC3669401

[btad610-B10] Gu Q , Nagaraj SH, Hudson NJ et al Genome-wide patterns of promoter sharing and co-expression in bovine skeletal muscle. BMC Genomics 2011;12:23. 21226902 10.1186/1471-2164-12-23 PMC3025955

[btad610-B11] Guo Y , Alexander K, Clark AG et al Integrated network analysis reveals distinct regulatory roles of transcription factors and microRNAs. RNA 2016;22:1663–72. 27604961 10.1261/rna.048025.114 PMC5066619

[btad610-B12] Hagberg A , Swart P, Chult DS. Exploring network structure, dynamics, and function using networkx. Technical report, Los Alamos National Lab. (LANL), Los Alamos, NM (United States), 2008.

[btad610-B13] Haury A-C , Mordelet F, Vera-Licona P et al TIGRESS: trustful inference of gene regulation using stability selection. BMC Syst Biol 2012;6:145. 23173819 10.1186/1752-0509-6-145 PMC3598250

[btad610-B14] Hew B , Tan QW, Goh W et al LSTrAP-crowd: prediction of novel components of bacterial ribosomes with crowd-sourced analysis of RNA sequencing data. BMC Biol 2020;18:114. 32883264 10.1186/s12915-020-00846-9 PMC7470450

[btad610-B15] Hicks SC , Okrah K, Paulson JN et al Smooth quantile normalization. Biostatistics 2018;19:185–98. 29036413 10.1093/biostatistics/kxx028 PMC5862355

[btad610-B16] Irrthum A , Wehenkel L, Geurts P et al Inferring regulatory networks from expression data using tree-based methods. PLoS One 2010;5:e12776. 20927193 10.1371/journal.pone.0012776 PMC2946910

[btad610-B17] Kanehisa M , Sato Y, Kawashima M et al KEGG as a reference resource for gene and protein annotation. Nucleic Acids Res 2016;44:D457–62. 26476454 10.1093/nar/gkv1070 PMC4702792

[btad610-B18] Kleinberg JM. Hubs, authorities, and communities. ACM Comput Surv 1999;31:5.

[btad610-B19] Kuijjer ML , Hsieh P-H, Quackenbush J et al lionessR: single sample network inference in R. BMC Cancer 2019a;19:1003. 31653243 10.1186/s12885-019-6235-7 PMC6815019

[btad610-B20] Kuijjer ML , Tung MG, Yuan G et al Estimating sample-specific regulatory networks. Iscience 2019b;14:226–40. 30981959 10.1016/j.isci.2019.03.021 PMC6463816

[btad610-B21] Kuijjer ML , Fagny M, Marin A et al PUMA: PANDA using microrna associations. Bioinformatics 2020;36:4765–73. 32860050 10.1093/bioinformatics/btaa571 PMC7750953

[btad610-B22] Lachmann A , Giorgi FM, Lopez G et al ARACNe-AP: gene network reverse engineering through adaptive partitioning inference of mutual information. Bioinformatics 2016;32:2233–5. 27153652 10.1093/bioinformatics/btw216 PMC4937200

[btad610-B23] Langfelder P , Horvath S. WGCNA: an R package for weighted correlation network analysis. BMC Bioinformatics 2008;9:559. 19114008 10.1186/1471-2105-9-559 PMC2631488

[btad610-B24] Lopes-Ramos CM , Kuijjer ML, Ogino S et al Gene regulatory network analysis identifies sex-linked differences in Colon cancer drug metabolism. Cancer Res 2018;78:5538–47. 30275053 10.1158/0008-5472.CAN-18-0454 PMC6169995

[btad610-B25] Lopes-Ramos CM , Chen C-Y, Kuijjer ML et al Sex differences in gene expression and regulatory networks across 29 human tissues. Cell Rep 2020;31:107795. 32579922 10.1016/j.celrep.2020.107795 PMC7898458

[btad610-B26] Lopes-Ramos CM , Belova T, Brunner TH et al Regulatory network of PD1 signaling is associated with prognosis in glioblastoma multiforme. Cancer Res 2021;81:5401–12. 34493595 10.1158/0008-5472.CAN-21-0730 PMC8563450

[btad610-B27] Marco A , Konikoff C, Karr TL et al Relationship between gene co-expression and sharing of transcription factor binding sites in *Drosophila melanogaster*. Bioinformatics 2009;25:2473–7. 19633094 10.1093/bioinformatics/btp462 PMC2752616

[btad610-B28] Meyer PE , Kontos K, Lafitte F et al Information-theoretic inference of large transcriptional regulatory networks. EURASIP J Bioinform Syst Biol 2007;2007:79879. 18354736 10.1155/2007/79879 PMC3171353

[btad610-B29] Mölder F , Jablonski KP, Letcher B et al Sustainable data analysis with snakemake. F1000Res 2021;10:33. 34035898 10.12688/f1000research.29032.1 PMC8114187

[btad610-B30] Nicolle R , Radvanyi F, Elati M. Coregnet: reconstruction and integrated analysis of co-regulatory networks. Bioinformatics 2015;31:3066–8. 25979476 10.1093/bioinformatics/btv305 PMC4565029

[btad610-B31] Paulson JN , Chen C-Y, Lopes-Ramos CM et al Tissue-aware RNA-seq processing and normalization for heterogeneous and sparse data. BMC Bioinformatics 2017;18:437–10. 28974199 10.1186/s12859-017-1847-x PMC5627434

[btad610-B32] Petralia F , Wang P, Yang J et al Integrative random Forest for gene regulatory network inference. Bioinformatics 2015;31:i197–205. 26072483 10.1093/bioinformatics/btv268 PMC4542785

[btad610-B33] Pierson E , Koller D, Battle A et al; GTEx Consortium. Sharing and specificity of co-expression networks across 35 human tissues. PLoS Comput Biol 2015;11:e1004220. 25970446 10.1371/journal.pcbi.1004220 PMC4430528

[btad610-B34] Reiss DJ , Plaisier CL, Wu W-J et al cMonkey2: automated, systematic, integrated detection of co-regulated gene modules for any organism. Nucleic Acids Res 2015;43:e87. 25873626 10.1093/nar/gkv300 PMC4513845

[btad610-B35] Ritchie ME , Phipson B, Wu D et al limma powers differential expression analyses for RNA-sequencing and microarray studies. Nucleic Acids Res 2015;43:e47. 25605792 10.1093/nar/gkv007 PMC4402510

[btad610-B36] Robinson MD , Oshlack A. A scaling normalization method for differential expression analysis of rna-seq data. Genome Biol 2010;11:R25–9. 20196867 10.1186/gb-2010-11-3-r25 PMC2864565

[btad610-B37] Saha A , Kim Y, Gewirtz AD et al; GTEx Consortium. Co-expression networks reveal the tissue-specific regulation of transcription and splicing. Genome Res 2017;27:1843–58. 29021288 10.1101/gr.216721.116 PMC5668942

[btad610-B38] Silverman EK , Schmidt HH, Anastasiadou E et al Molecular networks in network medicine: development and applications. Wiley Interdiscip Rev Syst Biol Med 2020;12:e1489. 32307915 10.1002/wsbm.1489 PMC7955589

[btad610-B39] Sonawane AR , Platig J, Fagny M et al Understanding tissue-specific gene regulation. Cell Rep 2017;21:1077–88. 29069589 10.1016/j.celrep.2017.10.001 PMC5828531

[btad610-B40] Stamatoyannopoulos JA , Snyder M, Hardison R et al; Mouse ENCODE Consortium. An encyclopedia of mouse dna elements (mouse encode). Genome Biol 2012;13:418–5. 22889292 10.1186/gb-2012-13-8-418 PMC3491367

[btad610-B41] Szklarczyk D , Gable AL, Lyon D et al String v11: protein–protein association networks with increased coverage, supporting functional discovery in genome-wide experimental datasets. Nucleic Acids Res 2019;47:D607–13. 30476243 10.1093/nar/gky1131 PMC6323986

[btad610-B42] Tosti L , Ashmore J, Tan BSN et al Mapping transcription factor occupancy using minimal numbers of cells in vitro and in vivo. Genome Res 2018;28:592–605. 29572359 10.1101/gr.227124.117 PMC5880248

[btad610-B43] Weinstein JN , Collisson EA, Mills GB et al; Cancer Genome Atlas Research Network. The cancer genome atlas pan-cancer analysis project. Nat Genet 2013;45:1113–20. 24071849 10.1038/ng.2764 PMC3919969

[btad610-B44] Zhao Y , Hou Y, Xu Y et al A compendium and comparative epigenomics analysis of cis-regulatory elements in the pig genome. Nat Commun 2021;12:2217. 33850120 10.1038/s41467-021-22448-x PMC8044108

